# Head and Neck Manifestations in Sarcoidosis: An All of Us Research Program Matched Case‐Control Study

**DOI:** 10.1002/oto2.70265

**Published:** 2026-06-16

**Authors:** Peter J. Attia, Sree R. Chinta, Raj Malhotra, Joseph Celidonio, Andrew R. Berman, Kenneth Yan, Rachel Kaye

**Affiliations:** ^1^ Department of Otolaryngology–Head and Neck Surgery Rutgers New Jersey Medical School Newark New Jersey USA; ^2^ Department of Medicine, Division of Pulmonary and Critical Care Medicine Rutgers New Jersey Medical School Newark New Jersey USA

**Keywords:** All of Us Research Program, cranial neuropathy, dysphagia, dysphonia, electronic medical records, epistaxis, multidisciplinary care, otolaryngology, sarcoidosis, sinonasal disease

## Abstract

**Objective:**

This study aims to assess the prevalence of head and neck (ENT) symptoms in patients with sarcoidosis.

**Study Design:**

1:4 matched‐case control study.

**Setting:**

Patients with and without sarcoidosis who enrolled in the All of Us Research Program and consented to electronic medical record use.

**Methods:**

Multivariate logistic regression examined the association between ENT symptoms and sarcoidosis, adjusting for autoimmune status, the Charlson Comorbidity Index score, and smoking status.

**Results:**

A total of 2446 patients with sarcoidosis were matched to 9783 controls. Compared to age‐, sex‐, race‐, and ethnicity‐matched controls and after adjustment, patients with sarcoidosis are more likely to have a recorded diagnosis of sinonasal symptoms, including chronic rhinitis (adjusted OR: 2.061 (95% CI: 1.779‐2.387), *P* < .001), chronic sinusitis (aOR: 1.735 (1.533‐1.964), *P* < .0001), and epistaxis (aOR: 1.479 (1.214‐1.801), *P* < .0001). Patients with sarcoidosis were more likely to have a recorded diagnosis of swallowing and airway symptoms, including dysphonia (aOR: 1.459 (1.215‐1.753), *P* < .0001) and dysphagia (aOR: 1.488 (1.312‐1.688), *P* < .0001). They were also more likely to have a recorded diagnosis of cranial nerve pathologies (aOR: 1.783 (1.525‐2.084), *P* < .0001) but less likely salivary gland pathologies (aOR: 0.600 (0.436‐0.827), *P* = .0018). Controls were more likely to have a recorded diagnosis of impacted cerumen (aOR: 0.505 (0.405‐0.630), *P* < .001).

**Conclusion:**

Patients with sarcoidosis are significantly more likely to be diagnosed with sinonasal symptoms, airway and swallowing symptoms, and cranial nerve pathologies with prevalence rates higher than previously reported. Sarcoidosis of the upper airway is likely underdiagnosed and consequently undertreated, necessitating greater awareness and further exploration of the etiology of upper airway symptoms in patients with sarcoidosis.

Sarcoidosis is a multisystemic inflammatory disorder with unknown etiology, characterized by noncaseating granulomas on pathology.^1^ While mainly associated with bilateral hilar adenopathy and pulmonary infiltration, sarcoidosis can be seen in many organ systems, including the skin, joints, eyes, liver, nervous system, and kidneys.^2^ The low prevalence of sarcoidosis makes large‐scale studies on its etiology, complications, and prognosis challenging. A Case‐Controlled Etiologic Study of Sarcoidosis (ACCESS) was the most extensive study to assess sarcoidosis and its multisystemic manifestations, enrolling 736 patients within 6 months of diagnosis.^3^ However, some symptoms may appear long after the initial diagnosis. There is a notable difficulty in diagnosing sarcoidosis and the organs involved, as its symptoms may mimic those of many underlying conditions.^1^

The head and neck manifestations in sarcoidosis are reported to occur in approximately 10% to 15% of patients.^4^ Ear, Nose, and Throat (ENT) manifestations range from sinonasal involvement, salivary gland enlargement, laryngeal masses, to cranial nerve dysfunction.^5^, ^6^ As ENT manifestations can be non‐specific, they may be linked to other chronic medical conditions with similar etiologies and thus may be under‐reported.^7^, ^8^, ^9^ Some patients experience spontaneous regression, while others develop refractory disease, underscoring the importance of accurate diagnosis for appropriate management.^2^

Large‐scale biobanks now enable population‐level assessment of associations between sarcoidosis and common ENT symptoms.^10^ Much of the current literature examines head and neck manifestations as initial presentations of sarcoidosis rather than co‐occurring clinical findings. This study attempts to bridge this knowledge gap by creating a case‐control trial using a nationwide cohort of patients with complete health histories and electronic medical records (EMR) data available for analysis and aims to determine whether patients with sarcoidosis are at a higher risk for ENT symptoms.

## Methods

### Database and Participant Selection

The All of Us Research Program comprises surveys, genomics, electronic health records, physical measurements, and wearables from a diverse nationwide population across the United States.^11^ The National Institute of Health's Institutional Review Board approved the All of Us Program. Using the All of Us Controlled Tier Dataset, all patients with EMR data were included. All patients documented to have been diagnosed with sarcoidosis were matched to nonsarcoidosis controls by race, ethnicity, sex at birth, and age at consent into the All of Us Research Program, with diagnosis codes listed in Supplemental Table S1, available online. The All of Us Research Program stores and recommends examining EMR data via Observational Medical Outcomes Partnership (OMOP) concept codes, which harmonize existing ICD and CPT codes entered by clinicians and health systems.^12^ Patients with EMR data were further stratified by a recorded diagnosis of sarcoidosis.

### Assessment of Head and Neck Symptoms and Diagnostic Pathways

Selected cases and controls had EMR data extracted for ENT diagnoses, chosen based upon review of the literature. Diagnoses of interest included laryngeal symptoms such as dysphonia, vocal cord paralysis, and dysphagia,^13^ as well as sinonasal symptoms including chronic rhinitis, chronic sinusitis, and epistaxis.^14^ Neurosarcoidosis is assessed by examining cranial nerve palsies.^5^ As such, we separately examined optic neuritis, facial nerve disease, trigeminal neuralgia, and acoustic nerve. As a proxy of CNVIII, we further examined vertigo and hearing loss. Various salivary gland pathologies have also been reported.^15^ Impacted cerumen was chosen as a negative control as it represents a common ENT diagnosis known not to be related to sarcoidosis. Supplemental Table S2, available online lists OMOP codes used to evaluate for these symptoms. Subject‐level records were searched for documented diagnoses; absence of a diagnosis code was assumed to indicate no diagnosis. It is noted that diagnosis codes may reflect clinician‐entered diagnosis codes following symptom assessments rather than procedurally confirmed disease. In post‐hoc analysis, we examine whether a patient underwent nasal endoscopy (OMOP concept ID 2106389) or laryngoscopy (OMOP concept IDs 4070239 or 4176717), both for the overall population and for those with dysphonia and chronic rhinitis or sinusitis.

### Assessment of Covariates

The Charlson Comorbidity Index (CCI) examines a variety of conditions, ranging from cardiovascular to hematological, and creates a validated score out of 33, predicting 10‐year mortality used as a surrogate for an individual's overall health status.^16^, ^17^, ^18^ As patients with sarcoidosis were reported to have been more likely to be diagnosed with autoimmune conditions, we also examined for the presence of autoimmune disorders in a separate analysis, defined as conditions that were related to OMOP concept ID 464621.^19^ Autoimmune diseases are not included in the CCI. We further identified whether patients were diagnosed with a head and neck, esophageal, or intrathoracic malignancy. Health insurance status was examined for all patients. Smoking status was based on self‐report of ≥100 cigarettes in a lifetime. Self‐reported annual income was also examined.

### Statistical Analysis

Propensity Score Matching (PSM) was used to match sarcoidosis cases to controls at a 1:4 ratio using nearest‐neighbor matching without replacement and a caliper width of 0.25. Exact matching was applied for sex. The propensity score was estimated using logistic regression, with sarcoidosis status as the outcome variable and sex, race, and ethnicity as predictors.^3^ The primary consent date was included to adjust for potential temporal confounding related to evolving enrollment patterns in the All of Us program. If a case was not matched to all four controls, they were kept in the analysis. Balance diagnostics were assessed before and after matching using Standardized Mean Differences (SMDs), with an SMD < 0.1 indicating adequate balance.

Subject demographics and the prevalence of ENT diagnosis were then tabulated and examined between cases and controls, reporting chi‐square *P*‐values. Unadjusted odds ratios were calculated, and adjusted odds ratios were calculated via multivariate logistic regression, adjusting for medical co‐morbidities (CCI score and autoimmune disease status) and smoking status. All odds ratios report 95% Wald Confidence Intervals. For all variables, subjects with missing smoking status were excluded from the adjusted analysis. Each ENT condition was analyzed independently; therefore, no correction for multiple comparisons was applied. A significance threshold of *P* < .05 was used for all statistical comparisons. All data extraction, manipulation, and analysis were completed in SAS 9.4.

## Results

### Subject Demographics

**Figure 1:**
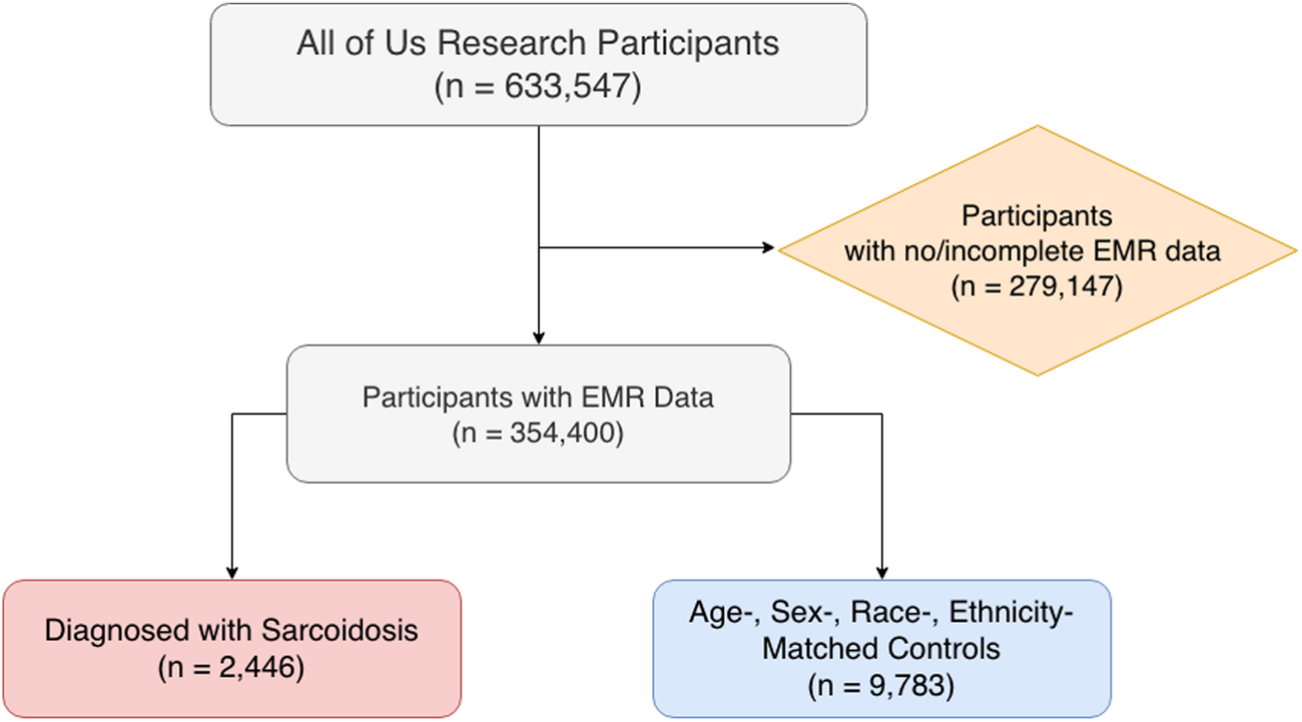
Patient inclusion flowchart. This image illustrates the patient inclusion and exclusion steps used to derive the final cohort used for this case‐control study.

633,547 patients were enrolled in the All of Us Program as of October 1, 2023, and 354,400 (55.9%) had EMR data. Of the patients with EMR data, 2446 (0.76%) were diagnosed with sarcoidosis. In matching for controls, 1 case only had 3 controls, and both the case and controls were retained. There are 9783 patients with no documented history of sarcoidosis (controls). The mean standardized difference improved from 0.69 to 0.0002, a 99.95% reduction, confirming appropriate balance. Figure 1 illustrates the selection of cases and controls.

Patients with sarcoidosis were more likely to have a longer length of EMR data compared to the controls (*P* < .0001), more likely to have been enrolled in the Research All of Us Program for a longer period (*P* < .0001), and less likely to have ever smoked cigarettes as compared to the controls (*P* < .05). Many patients (both sarcoidosis and controls) were more likely to be in a lower income group, with no difference between the two groups. Sarcoidosis patients were more likely to be insured by Government‐Sponsored plans (Medicare, Medicaid, Military, Veterans Affairs) or have unknown insurance status, while controls were more likely to be under a private insurance plan or uninsured (*P* < .0001). The 2 groups were equivalent by race, ethnicity, sex, and age at consent in the All of Us Program (Table 1).

**Table 1:** Demographics

Variable & Category	Sarcoidosis (n = 2446)	Control (n = 9783)	*P* value
Biological sex at birth			1.0000
Female (N [%])	1621 (66.27%)	6484 (66.28%)	
Male (N [%])	798 (32.62%)	3191 (32.62%)	
Other (N [%])	27 (1.1%)	108 (1.1%)	
Race			.9998
Black (N [%])	855 (34.96%)	3418 (34.94%)	
Other (N [%])	401 (16.39%)	1605 (16.41%)	
White (N [%])	1190 (48.65%)	4760 (48.66%)	
Ethnicity			.9980
Hispanic or Latino (N [%])	207 (8.46%)	824 (8.42%)	
Non‐Hispanic (N [%])	2174 (88.88%)	8699 (88.92%)	
Other (N [%])	65 (2.66%)	260 (2.66%)	
Age at Study Consent for All of US (Median [IQR])			.2255
18‐44	171 (6.99%)	735 (7.51%)	
45‐64	912 (37.3%)	3782 (38.7%)	
65+	1363 (55.7%)	5266 (53.8%)	
Length of time in All of Us (years‐median [IQR])	11 (7‐17)	7 (4‐12)	**<.0001**
Length of time of EMR follow‐up (years‐median [IQR])	14 (8‐20)	9 (4‐16)	**<.0001**
Smoking status			**.0469**
Never smoker	1375 (56.2%)	5264 (53.8%)	
Smoker (past or current)	997 (40.8%)	4186 (42.8%)	
Unknown	74 (3.03%)	333 (3.40%)	
Insurance status			
Private‐employer‐based, individual, or other	878 (35.9%)	3555 (36.3%)	**<.0001**
Government sponsored	1209 (49.4%)	4699 (48.0%)	
Uninsured	51 (2.09%)	476 (4.87%)	
Unknown	308 (12.6%)	1053 (10.76%)	
Self‐reported annual income			
<$25,000	625 (31.6%)	2523 (32.7%)	.1113
$25,000‐$50,000	381 (19.3%)	1359 (17.6%)	
$50,000‐$100,000	488 (23.7%)	1829 (18.7%)	
$100,000‐$150,000	247 (10.1%)	939 (9.6%)	
>$150,000	236 (9.7%)	1059 (10.8%)	
Unknown	469 (19.2%)	2074 (21.20%)	

Values in bold indicate statistical significance (*P* < .05).

### Medical Covariates

Medical covariates were assessed by the presence of any autoimmune diseases and by the broader CCI (Table 2). Patients with sarcoidosis were more likely to have a recorded diagnosis of any autoimmune condition (*P* < .0001). Using the CCI, we found that the sarcoidosis group was significantly more likely to be diagnosed with illnesses in all categories except for AIDS‐defining illness. Patients with sarcoidosis are more likely to have a documented diagnosis of multiple medical co‐morbidities compared to controls (*P* < .0001).

**Table 2:** Medical Covariates and CCI Scores Stratified by Diagnosis of Sarcoidosis

	Sarcoidosis (n = 2446)	Control (n = 9783)	*P* value
Charlson Comorbidity Index	N (%)	N (%)	
Congestive heart failure	842 (34.42%)	1124 (11.49%)	**<.0001**
Dementia	95 (3.88%)	238 (2.43%)	**<.0001**
Diabetes mellitus with complications	972 (39.74%)	2526 (25.82%)	**<.0001**
Hemiplegia	273 (11.16%)	462 (4.72%)	**<.0001**
HIV/AIDS	49 (2%)	196 (2%)	.9995
Malignancy (including leukemia or lymphoma)	1451 (59.32%)	3739 (38.22%)	**<.0001**
Metastatic solid tumor	821 (33.57%)	1930 (19.73%)	**<.0001**
Mild liver disease	777 (31.77%)	1560 (15.95%)	**<.0001**
Moderate/severe liver disease	62 (2.53%)	86 (0.88%)	**<.0001**
Chronic pulmonary disease	1538 (62.88%)	3140 (32.1%)	**<.0001**
Renal disease	1169 (47.79%)	2491 (25.46%)	**<.0001**
Rheumatic disease	609 (24.9%)	726 (7.42%)	**<.0001**
CCI total score (median [IQR])	6 (3‐9)	2 (0‐6)	**<.0001**
Known diagnosis of autoimmune illnesses (N [%])	644 (26.33%)	874 (8.93%)	**<.0001**

Values in bold indicate statistical significance (*P* < .05).

### Descriptive Analysis of ENT Symptoms

Of patients with sarcoidosis, 1309 (53.5%) were documented with at least ENT symptom, significantly higher than the 2729 (27.9%) of controls (*P* < .0001). The median number of diagnoses was 1 (IQR: 0‐2) in the sarcoidosis group and 0 (IQR: 0‐1) within the controls, a statistically significant difference (*P* < .0001). Patients with sarcoidosis had higher prevalence for all conditions except for salivary gland pathologies and impacted cerumen. The control group was more likely to have a documented diagnosis of impacted cerumen (*P* = .0004). Individual prevalences are shown in Table 3. A subgroup analysis examining specific cranial nerve pathologies (optic, trigeminal, facial, acoustic) reflects that patients with Sarcoidosis are more likely to have a documented diagnosis of these pathologies (Supplemental Table S3, available online).

**Table 3:** ENT Diagnosis Stratified by Sarcoidosis Diagnosis

	Sarcoidosis (n = 2446)	Controls (n = 9783)	
Diagnosis	# of Patients with symptoms [N (%)]	# of Patients with symptoms [N (%)]	*P* value
Dysphagia	574 (23.47%)	1031 (10.54%)	**<.0001**
Dysphonia	234 (9.57%)	383 (3.91%)	**<.0001**
Chronic rhinitis	398 (16.27%)	566 (5.79%)	**<.0001**
Chronic sinusitis	557 (22.77%)	997 (10.19%)	**<.0001**
Vocal cord paralysis	50 (2.04%)	59 (0.6%)	**<.0001**
Salivary gland pathologies	55 (2.25%)	241 (2.46%)	.5878
Cranial nerve pathologies	352 (14.4%)	574 (5.87%)	**<.0001**
Epistaxis	196 (8.01%)	327 (3.34%)	**<.0001**
Impacted cerumen	106 (4.33%)	607 (6.20%)	**.0004**

Values in bold indicate statistical significance (*P* < .05).

### Odds of ENT Symptoms with Sarcoidosis

In the unadjusted model, patients with sarcoidosis had significantly higher odds of dysphonia, dysphagia, chronic rhinitis, chronic sinusitis, vocal cord paralysis, cranial nerve pathology, and epistaxis compared with controls. However, salivary gland pathology was similar between groups. All individual cranial nerve pathologies also showed increased odds in the sarcoidosis population (Supplemental Table S3, available online). Impacted cerumen had significantly higher odds of diagnosis in the controls (Table 4).

**Table 4:** Unadjusted and Adjusted Odds Ratio of ENT Symptoms

Symptoms	Unadjusted odds ratios [OR (95% Wald CI)]	*P*	Adjusted (autoimmune/smoking) odds ratios [OR (95% Wald CI)]	*P*	Adjusted (CCI score/smoking) odds ratios OR (95% Wald CI)]	*P*
Dysphagia	2.603 (2.323‐2.916)	**<.0001**	2.123 (1.883‐2.395)	**<.0001**	1.488 (1.312‐1.688)	**<.0001**
Dysphonia	2.596 (2.192‐3.075)	**<.0001**	2.102 (1.758‐2.513)	**<.0001**	1.459 (1.215‐1.753)	**<.0001**
Chronic rhinitis	3.165 (2.76‐3.629)	**<.0001**	2.662 (2.306‐3.074)	**<.0001**	2.061 (1.779‐2.387)	**<.0001**
Chronic sinusitis	2.598 (2.316‐2.915)	**<.0001**	2.171 (1.924‐2.450)	**<.0001**	1.735 (1.533‐1.964)	**<.0001**
Vocal cord paralysis	3.439 (2.353‐5.026)	**<.0001**	2.681 (1.785‐4.025)	**<.0001**	1.443 (0.955‐2.181)	.0818
Salivary gland pathologies	0.911 (0.677‐1.225)	.5364	0.662 (0.484‐0.905)	**.0097**	0.600 (0.436‐0.827)	**.0018**
Cranial nerve pathologies	2.698 (2.342‐3.105)	**<.0001**	2.300 (1.976‐2.677)	**<.0001**	1.817 (1.565‐2.110)	**<.0001**
Epistaxis	2.519 (2.098‐3.025)	**<.0001**	2.123 (1.750‐2.575)	**<.0001**	1.479 (1.214‐1.801)	**.0001**
Impacted cerumen	0.685 (0.555‐0.846)	**.0004**	0.636 (0.512‐0.792)	**<.0001**	0.505 (0.405‐0.630)	**<.0001**

Values in bold indicate statistical significance (*P* < .05).

Adjusted models exclude 407 patients (74 [3.03%] cases and 303 controls [3.4%]) due to missing smoking status. The first model assessed the relationship between these manifestations with sarcoidosis while controlling for both autoimmune illnesses and smoking status. In this analysis, associations remained, although attenuated, compared with the unadjusted model. Salivary gland pathologies were also less likely to be diagnosed in the sarcoidosis population than in the controls (OR: 0.662 (0.484‐0.905), *P* = .0097). Impacted cerumen was also strengthened towards being protective in the group of patients with sarcoidosis (OR: 0.636 (0.512‐0.792), *P* < .0001).

The second model adjusted for CCI scores as a continuous variable along with smoking status. These findings were similar to our first model and with findings being even more attenuated then prior, except that vocal cord paralysis was no longer statistically significant but remained a trend towards being diagnosed at a higher prevalence in the sarcoidosis population. We utilized this model for our subgroup analysis of individual cranial nerves, and we report that patients with sarcoidosis were more likely to have trigeminal and facial nerve palsies as well as vertigo (Supplemental Table S3, available online). A sensitivity analysis examining each individual CCI component showed that all ENT manifestations—except salivary gland pathologies and impacted cerumen—remained more likely to be diagnosed in the sarcoidosis group, consistent with the primary findings (Supplemental Table S4, available online).

### Post‐Hoc Analysis of Nasal Endoscopy and Laryngoscopy

236 (9.7%) patients with sarcoidosis and 362 (3.7%) controls had undergone a laryngoscopy, with patients with sarcoidosis more likely to undergo a laryngoscopy (*P* < .0001). Likewise, 187 (7.7%) patients with sarcoidosis and 276 (2.8%) controls had undergone a nasal endoscopy, with patients with sarcoidosis being more likely to undergo a nasal endoscopy (*P* < .0001).^20^ Of the 617 patients with dysphonia, only 190 (30.8%) had a documented laryngoscopy, and of the 109 patients with vocal cord paralysis, only 35 (32%) had a documented laryngoscopy. Only 11% of sarcoidosis patients (n = 27) and 11% of controls (n = 41) who had undergone a laryngoscopy had a history or diagnosis of either malignancy of the pharynx, larynx, esophagus, or intrathoracic cavity. Only 17% of patients across this cohort with these malignancies are undergoing laryngoscopy. Similarly, of the 2,015 patients diagnosed with chronic rhinitis and/or sinusitis, only 356 (17.7%) had any type of nasal endoscopy documented.

## Discussion

This is one of the first studies to examine ENT prevalence in sarcoidosis using biobank data, as other are limited to case series or local institutional data. We note a higher prevalence of sarcoidosis compared to national estimates and a higher prevalence of ENT conditions in the sarcoidosis cohort than in the controls.^3^, ^21^ Our findings revealed an increased prevalence of dysphonia, dysphagia, chronic rhinitis, chronic sinusitis, cranial nerve pathologies, and epistaxis in unadjusted and adjusted analyses. Salivary gland pathologies were less common in the sarcoidosis population in adjusted analysis, whereas vocal cord paralysis lost statistical significance in the adjusted analysis. Otolaryngologists play a unique role in the diagnostic evaluation of upper aerodigestive complaints of unknown origin; therefore, familiarity with sarcoidosis‐related manifestations may help broaden the differential diagnosis, guide targeted evaluation, and facilitate timely multidisciplinary collaboration.

In an examination of demographics, we observed a statistically significant difference in insurance status distribution between the sarcoidosis and control cohorts (*χ* ^2^ = 41.88, *P* < .0001). Although statistically significant, absolute differences across insurance categories were small. We note a higher percentage of patients with government‐based insurance in the sarcoidosis group, potentially because 53.8% of sarcoidosis patients were ≥65 years old.

Examination of sinonasal symptoms, chronic rhinitis, chronic sinusitis, and epistaxis, reveals an increased prevalence in the sarcoidosis cohort.^6^ Chronic rhinitis was included to replicate Reed et al's model for sinonasal sarcoidosis.^22^ Chronic sinusitis was chosen as it has a feature of bilateral nasal obstruction.^23^ Epistaxis, though less common, was also associated with sinonasal sarcoidosis.^24^ Reports of sinonasal sarcoidosis typically report a prevalence of 1% to 6.5%, although one study reported a rate as high as 30% in referrals to an otolaryngology practice among sarcoidosis patients.^25^ In this study, 12% of patients with sarcoidosis were diagnosed with two or more sinonasal symptoms, higher than previously noted.^26^ Future studies should further clarify sinonasal‐sarcoidosis associations.

In sarcoidosis, laryngeal manifestations may be observed by symptoms of dysphonia, vocal cord paralysis, or dysphagia. Dysphonia has been reported to be caused by compression of the recurrent laryngeal nerve from adenopathy,^27^ neurosarcoidosis of the vagus nerve,^28^ or edema and inflammation affecting the larynx or vocal cords.^29^ Dysphonia is associated with a large range of etiologies, some of which were elevated in the sarcoidosis population, such as cancer and COPD.^20^ However, even after controlling for these co‐morbidities, we still report an increased likelihood of dysphonia within our sarcoidosis cohort. Likewise, vocal cord paralysis may have been insignificant after adjustment due to sample size limitations, amplifying concerns for underreporting in our cohort. Dysphagia arises from diverse etiologies, but in sarcoidosis, dysphagia is mostly secondary to laryngeal involvement with direct laryngeal inflammation and infiltration, causing distortion of the supraglottis.^30^ Prior estimates report a prevalence between 0.5% and 8.3% in laryngeal sarcoidosis.^31^ This study found 23% of patients with sarcoidosis were diagnosed with dysphagia, potentially affirming that there is an underdiagnosis of these etiologies.^32^

Examination of procedural data revealed that, while patients with sarcoidosis were more likely to undergo endoscopic evaluation, the overall rates of nasal endoscopy among patients with chronic rhinosinusitis and of laryngoscopy among patients with dysphonia remained low. This may suggest under‐referral to otolaryngology for guideline‐recommended diagnostic evaluation. However, as not all patients with upper body malignancy, vocal cord paralysis, or dysphonia underwent laryngoscopy, incomplete procedural capture within the EMR may also contribute to these findings. In the absence of provider specialty data, we are unable to determine what is the true cause of our findings.

In this study, 14.3% of patients with sarcoidosis were diagnosed with cranial nerve pathologies, higher than the 2.5% observed in a southern European population conducted by Ramos‐Casias et al.^28^ This difference may be due to methodological differences and population inclusion criteria. After adjusting for comorbidities, the association between sarcoidosis and cranial nerve pathologies was attenuated, suggesting non‐sarcoidosis factors may contribute. In further examination of specific cranial nerve pathologies, we report mixed findings: some pathologies were more likely to be diagnosed after adjustment (optic, trigeminal, or facial) than others (acoustic and sensorineural hearing loss). Both optic and facial nerve palsies are the most common in this population and the population studied by Ramos‐Casias et al.

Prevalence of salivary gland pathologies in sarcoidosis varies widely throughout the literature, but can be as high as 5% to 10% of patients.^6^ Any salivary gland pathology was included due to reports of variable presentations.^24^ Unlike the other symptoms, we found no difference between the sarcoidosis group and the controls, and the prevalence rates were similar to a previous cohort studying the difference in sarcoidosis manifestations in men and women.^33^ Our finding that sarcoidosis patients are less likely to be diagnosed with salivary gland pathologies is novel and raises questions about whether sarcoidosis affects salivary glands differently than other systemic diseases or if underreporting plays a role. Future studies should explore potential immunologic factors and diagnostic biases to clarify this novel finding.

Impacted cerumen was chosen as a negative control, and its inverse association with sarcoidosis supports the specificity of our findings. This further supports that the findings observed are less likely due to increased healthcare utilization within the sarcoidosis population and more likely to reflect true disease burden.^34^

### Limitations

As with any analysis of EMR data, findings are limited based on physician entry of diagnosis codes, a reporting bias. The diagnosis codes entered may not have been entered following gold standard evaluation (ie, laryngoscopy and nasal endoscopy in the setting of dysphonia, vocal cord paralysis, and rhinitis), and we are unable to determine the specialty of the provider who entered the codes. Notably, only a minority of patients diagnosed with vocal cord paralysis had documented laryngoscopy, likely reflecting incomplete procedural capture rather than the absence of diagnostic evaluation, as laryngoscopy is required to establish this diagnosis. We cannot determine, with certainty, when patients were first diagnosed with sarcoidosis. Due to the limitations in sample size, we are unable to differentiate between unilateral and bilateral vocal cord paralysis, or examine nasal obstruction.^35^ Furthermore, we cannot definitively determine if symptoms were attributable to sarcoidosis, as sarcoidosis is a diagnosis of exclusion, and symptoms may be related to other conditions, such as allergies. *All of Us* does not have this type of granular data accessible to researchers. Despite PSM and adjustments, residual confounding from comorbid conditions may persist.

## Conclusion

Sarcoidosis manifests with a diverse range of clinical manifestations. Leveraging a large biobank dataset, this study demonstrates that ENT diagnoses, such as dysphonia, dysphagia, chronic rhinitis, chronic sinusitis, cranial nerve pathologies, and epistaxis, are more likely to occur in this population, even after controlling for behavioral and medical co‐morbidities. Further, we suspect that ENT manifestations are not only under‐reported but also under‐diagnosed, underscoring the role of otolaryngologists in the multidisciplinary care of sarcoidosis and the need for heightened awareness and early intervention to improve patient outcomes. Our findings affirm the need for prospective studies to assess the phenomenon of ENT pathologies in patients with sarcoidosis.

## Author Contributions

**Peter J. Attia**, design/conceptualization, methodology, data curation, data acquisition, formal analysis, software, investigation, writing—original draft, writing—revising and editing; **Sree R. Chinta**, design/conceptualization, writing—revising and editing; **Raj Malhotra**, design/conceptualization, writing—revising and editing; **Joseph Celidonio**, design/conceptualization, writing—revising and editing; **Andrew R. Berman**, design/conceptualization, methodology, writing—revising and editing; **Kenneth Yan**, supervision, methodology, design/conceptualization, investigation, writing—revising and editing; **Rachel Kaye**, supervision, design/conceptualization, methodology, investigation, writing—revising and editing.

## Disclosures

### Competing interests

None.

### Funding source

None.

## Supporting information

Supplemental Table 1. Listing of Sarcoidosis Diagnosis Codes. This table lists all the Observational Medical Outcomes Partnership (OMOP) and Systematized Nomenclature of Medicine Clinical Terms (SNOMED) concept codes used to identify patients with sarcoidosis.

Supplemental Table 2. Listing of ENT Diagnosis Codes. This table lists all the Observational Medical Outcomes Partnership (OMOP) and Systematized Nomenclature of Medicine Clinical Terms (SNOMED) concept codes used to identify the ENT manifestations examined in this study.

Supplemental Table 3. Prevalence and Association of Cranial Nerve and Audiovestibular Disorders. Shows OMOP and SNOMED codes, cohort prevalence, and unadjusted and adjusted regression for cranial nerve and audiovestibular disorders in sarcoidosis and controls.

Supplemental Table 4. Sensitivity analysis: adjusted odds ratios of ENT diagnoses, controlling individually for Charlson Comorbidity Index elements. Matrix of adjusted odds ratios for ENT diagnoses, with sensitivity analyses controlling individually for each Charlson Comorbidity Index component.
