# Supplementary material for: Head and Neck Manifestations in Sarcoidosis: An All of Us Research Program Matched Case‐Control Study
Source: OTO Open. 2026 Jun 16;10(2):e70265. doi: 10.1002/oto2.70265 (PMC13270404; doi:10.1002/oto2.70265)
Supplement: Supplementary file 1 — Supplemental Table 1. Listing of Sarcoidosis Diagnosis Codes. This table lists all the Observational Medical Outcomes Partnership (OMOP) and Systematized Nomenclature of Medicine Clinical Terms (SNOMED) concept codes used to identify patients with sarcoidosis. [file OTO2-10-e70265-s002.docx]

Supplemental Table 1. Listing of Sarcoidosis Diagnosis Codes

| Name | Concept Id | Source  Standard | Vocab | Code |
| --- | --- | --- | --- | --- |
| Sarcoidosis | 438688 | Standard | SNOMED | 31541009 |
| Pulmonary sarcoidosis | 4086243 | Standard | SNOMED | 24369008 |
| Sarcoidosis of lung with sarcoidosis of lymph nodes | 4093002 | Standard | SNOMED | 187233002 |
| Cardiac sarcoidosis | 4326751 | Standard | SNOMED | 75403004 |
| Sarcoid heart muscle disease | 4111541 | Standard | SNOMED | 195033009 |
| Cutaneous sarcoidosis | 4185547 | Standard | SNOMED | 55941000 |
| Lymph node sarcoidosis | 4274184 | Standard | SNOMED | 64757003 |
| Sarcoid arthropathy | 4262578 | Standard | SNOMED | 361197009 |
| Anterior uveitis due to sarcoidosis | 606435 | Standard | SNOMED | 1144978007 |
| Sarcoid iridocyclitis | 45772123 | Standard | SNOMED | 352941000119102 |
| Sarcoid uveitis | 4190954 | Standard | SNOMED | 415359008 |
| Myositis in sarcoidosis | 4002813 | Standard | SNOMED | 203042003 |
| Sarcoid myopathy | 4105025 | Standard | SNOMED | 193251003 |
| Sarcoid meningitis | 440699 | Standard | SNOMED | 192673008 |
| Multiple cranial nerve palsies in sarcoidosis | 4105005 | Standard | SNOMED | 193101001 |
| Sarcoidosis, lupus pernio type | 4216556 | Standard | SNOMED | 72470008 |
| Granulomatous sarcoid nephropathy | 4290339 | Standard | SNOMED | 37061001 |
| Subcutaneous sarcoidosis | 4214896 | Standard | SNOMED | 80941006 |
| Lofgrens syndrome | 4081069 | Standard | SNOMED | 238676008 |
| Ocular sarcoidosis | 4123081 | Standard | SNOMED | 234526006 |
| Restrictive cardiomyopathy secondary to sarcoidosis | 4191606 | Standard | SNOMED | 39041004 |
| Sarcoid arthritis | 4263646 | Standard | SNOMED | 361198004 |
| Sarcoid neuropathy | 4105018 | Standard | SNOMED | 193195000 |
| Sarcoidosis of digestive system | 42536513 | Standard | SNOMED | 735433009 |
| Sarcoidosis, Darier-Roussy type | 4072323 | Standard | SNOMED | 21787007 |
| Sarcoidosis-induced erythema nodosum | 4033215 | Standard | SNOMED | 238675007 |
| Stage 3 pulmonary sarcoidosis | 4119448 | Standard | SNOMED | 233769008 |
