# Supplementary material for: Head and Neck Manifestations in Sarcoidosis: An All of Us Research Program Matched Case‐Control Study
Source: OTO Open. 2026 Jun 16;10(2):e70265. doi: 10.1002/oto2.70265 (PMC13270404; doi:10.1002/oto2.70265)
Supplement: Supplementary file 2 — Supplemental Table 2. Listing of ENT Diagnosis Codes. This table lists all the Observational Medical Outcomes Partnership (OMOP) and Systematized Nomenclature of Medicine Clinical Terms (SNOMED) concept codes used to identify the ENT manifestations examined in this study. [file OTO2-10-e70265-s003.docx]

Supplemental Table 2. Listing of ENT Diagnosis Codes

| Head and Neck Symptom Group | Name | Concept Id | Source/Standard | Vocab | Code |
| --- | --- | --- | --- | --- | --- |
| Chronic Rhinitis | Atrophic rhinitis | [4101701](https://databrowser.researchallofus.org/ehr/conditions/4101701) | Standard | SNOMED | 28111002 |
|  | Chronic atrophic rhinitis | [4110489](https://databrowser.researchallofus.org/ehr/conditions/4110489) | Standard | SNOMED | 195769008 |
|  | Chronic rhinitis | [259848](https://databrowser.researchallofus.org/ehr/conditions/259848) | Standard | SNOMED | 86094006 |
|  | Non-allergic rhinitis | [42872416](https://databrowser.researchallofus.org/ehr/conditions/42872416) | Standard | SNOMED | 311000119101 |
|  | Ulcerative rhinitis | [4270705](https://databrowser.researchallofus.org/ehr/conditions/4270705) | Standard | SNOMED | 36669007 |
| Chronic Sinusitis | Chronic bilateral maxillary sinusitis | [761761](https://databrowser.researchallofus.org/ehr/conditions/761761) | Standard | SNOMED | 15981911000119100 |
|  | Chronic ethmoidal sinusitis | [132932](https://databrowser.researchallofus.org/ehr/conditions/132932) | Standard | SNOMED | 73237007 |
|  | Chronic frontal sinusitis | [139841](https://databrowser.researchallofus.org/ehr/conditions/139841) | Standard | SNOMED | 60130002 |
|  | Chronic frontoethmoidal sinusitis | [4051488](https://databrowser.researchallofus.org/ehr/conditions/4051488) | Standard | SNOMED | 232397007 |
|  | Chronic left maxillary sinusitis | [761762](https://databrowser.researchallofus.org/ehr/conditions/761762) | Standard | SNOMED | 15981991000119100 |
|  | Chronic panethmoidal sinusitis | [4051487](https://databrowser.researchallofus.org/ehr/conditions/4051487) | Standard | SNOMED | 232395004 |
|  | Chronic recurrent sinusitis | [4179673](https://databrowser.researchallofus.org/ehr/conditions/4179673) | Standard | SNOMED | 427909005 |
|  | Chronic right maxillary sinusitis | [765276](https://databrowser.researchallofus.org/ehr/conditions/765276) | Standard | SNOMED | 15981951000119100 |
|  | Chronic sinusitis | [257012](https://databrowser.researchallofus.org/ehr/conditions/257012) | Standard | SNOMED | 40055000 |
|  | Chronic sphenoidal sinusitis | [134661](https://databrowser.researchallofus.org/ehr/conditions/134661) | Standard | SNOMED | 38961000 |
| Dysphagia | Constant low-grade dysphagia | [4278990](https://databrowser.researchallofus.org/ehr/conditions/4278990) | Standard | SNOMED | 65191003 |
|  | Dysphagia | [31317](https://databrowser.researchallofus.org/ehr/conditions/31317) | Standard | SNOMED | 40739000 |
|  | Esophageal dysphagia | [4254223](https://databrowser.researchallofus.org/ehr/conditions/4254223) | Standard | SNOMED | 40890009 |
|  | Functional dysphagia | [36716717](https://databrowser.researchallofus.org/ehr/conditions/36716717) | Standard | SNOMED | 722875003 |
|  | Intermittent dysphagia | [4055360](https://databrowser.researchallofus.org/ehr/conditions/4055360) | Standard | SNOMED | 19597002 |
|  | Oral phase dysphagia | [26823](https://databrowser.researchallofus.org/ehr/conditions/26823) | Standard | SNOMED | 429975007 |
|  | Oropharyngeal dysphagia | [440530](https://databrowser.researchallofus.org/ehr/conditions/440530) | Standard | SNOMED | 71457002 |
|  | Pharyngeal dysphagia | [45757559](https://databrowser.researchallofus.org/ehr/conditions/45757559) | Standard | SNOMED | 21101000119105 |
| Dysphonia | Difficulty talking | [4114720](https://databrowser.researchallofus.org/ehr/conditions/4114720) | Standard | SNOMED | 286378009 |
|  | Dysphonia | [40326053](https://databrowser.researchallofus.org/ehr/conditions/40326053) | Standard | SNOMED | 16617009 |
| Epistaxis | Bleeding from nose | [4096682](https://databrowser.researchallofus.org/ehr/conditions/4096682) | Standard | SNOMED | 249366005 |
| Vocal Cord Paralysis | Bilateral partial vocal cord paralysis | [260130](https://databrowser.researchallofus.org/ehr/conditions/260130) | Standard | SNOMED | 195844002 |
|  | Complete bilateral paralysis of vocal cords | [260135](https://databrowser.researchallofus.org/ehr/conditions/260135) | Standard | SNOMED | 42655007 |
|  | Complete paralysis of left vocal cord | [37206934](https://databrowser.researchallofus.org/ehr/conditions/37206934) | Standard | SNOMED | 787603009 |
|  | Complete paralysis of right vocal cord | [37206935](https://databrowser.researchallofus.org/ehr/conditions/37206935) | Standard | SNOMED | 787604003 |
|  | Paralysis of left vocal cord | [35608079](https://databrowser.researchallofus.org/ehr/conditions/35608079) | Standard | SNOMED | 764724007 |
|  | Paralysis of right vocal cord | [35608081](https://databrowser.researchallofus.org/ehr/conditions/35608081) | Standard | SNOMED | 764726009 |
|  | Paresis of left vocal cord | [605317](https://databrowser.researchallofus.org/ehr/conditions/605317) | Standard | SNOMED | 1052239007 |
|  | Paresis of right vocal cord | [605318](https://databrowser.researchallofus.org/ehr/conditions/605318) | Standard | SNOMED | 1052240009 |
|  | Vocal cord paralysis | [261599](https://databrowser.researchallofus.org/ehr/conditions/261599) | Standard | SNOMED | 302912005 |
| Salivary Gland Pathologies | Salivary gland finding | [4158189](https://databrowser.researchallofus.org/ehr/conditions/4158189) | Standard | SNOMED | 271612000 |
| Cranial Nerve Pathologies/  Neurosarcoidosis | Cranial nerve disorder | [441848](https://databrowser.researchallofus.org/ehr/conditions/441848) | Standard | SNOMED | 73013002 |
|  | Optic nerve disorder | 374360 | Standard | SNOMED | 77157004 |
|  | Trigeminal nerve disorder | 440703 | Standard | SNOMED | 64309007 |
|  | Facial nerve disorder | 378135 | Standard | SNOMED | 422426003 |
|  | Acoustic nerve disorder | 439842 | Standard | SNOMED | 77949003 |
|  | Sensorineural Hearing loss | 377889 | Standard | SNOMED | 15188001 |
|  | Vertigo | 439383 | Standard | SNOMED | 399153001 |
| Impacted Cerumen | Impacted cerumen | 374375 | Standard | SNOMED | 18070006 |
|  | Impacted cerumen of bilateral ears | 760047 | Standard | SNOMED | 1083241000119110 |
|  | Impacted cerumen in left ear | 760147 | Standard | SNOMED | 1088371000119100 |
|  | Impacted cerumen in right ear | 760186 | Standard | SNOMED | 1090981000119100 |
