# Supplementary material for: Head and Neck Manifestations in Sarcoidosis: An All of Us Research Program Matched Case‐Control Study
Source: OTO Open. 2026 Jun 16;10(2):e70265. doi: 10.1002/oto2.70265 (PMC13270404; doi:10.1002/oto2.70265)
Supplement: Supplementary file 3 — Supplemental Table 3. Prevalence and Association of Cranial Nerve and Audiovestibular Disorders. Shows OMOP and SNOMED codes, cohort prevalence, and unadjusted and adjusted regression for cranial nerve and audiovestibular disorders in sarcoidosis and controls. [file OTO2-10-e70265-s004.docx]

**Supplemental Table 3. Prevalence and Association of Cranial Nerve and Audiovestibular Disorders**

| **Outcome** | **OMOP  Concept ID** | **SNOMED CT** | **Sarcoidosis  (n = 2446)** | **Control  (n = 9783)** | **Unadjusted Odds (95% CI)** | **Unadj  p-value** | **Adjusted OR  (95% CI)** | **Adj  p-value** |
| --- | --- | --- | --- | --- | --- | --- | --- | --- |
| **Optic nerve disorder** | 374360 | 77157004 | 308 (12.6%) | 598 (6.1%) | 2.14 (1.55 – 2.94) | < .001 | 1.16 (0.99 - 1.36) | 0.066 |
| **Trigeminal nerve disorder** | 440703 | 64309007 | 58 (2.4%) | 110 (1.1%) | 2.90 (2.19 – 3.83 | < .001 | 1.47 (1.05 - 2.06) | 0.025 |
| **Facial nerve disorder** | 378135 | 422426003 | 87 (3.6%) | 123 (1.3%) | 1.96 (1.16 – 3.31) | < .001 | 1.80 (1.34 - 2.42) | < .001 |
| **Acoustic nerve disorder** | 439842 | 77949003 | 21 (0.9%) | 43 (0.4%) | 1.86 (1.67 – 2.07) | < .001 | 1.27 (0.74 - 2.19) | 0.383 |
| **Sensorineural Hearing loss** | 377889 | 15188001 | 459 (18.7%) | 1112 (11.4%) | 2.37 (1.65 – 3.39) | < .001 | 1.08 (0.95 - 1.23) | 0.225 |
| **Vertigo** | 439383 | 399153001 | 48 (2.0%) | 82 (0.8%) | 1.80 (1.60 – 2.03) | < .001 | 1.48 (1.01 - 2.18) | 0.047 |
