# Supplementary material for: Head and Neck Manifestations in Sarcoidosis: An All of Us Research Program Matched Case‐Control Study
Source: OTO Open. 2026 Jun 16;10(2):e70265. doi: 10.1002/oto2.70265 (PMC13270404; doi:10.1002/oto2.70265)
Supplement: Supplementary file 4 — Supplemental Table 4. Sensitivity analysis: adjusted odds ratios of ENT diagnoses, controlling individually for Charlson Comorbidity Index elements. Matrix of adjusted odds ratios for ENT diagnoses, with sensitivity analyses controlling individually for each Charlson Comorbidity Index component. [file OTO2-10-e70265-s001.docx]

Supplemental Table 4. Sensitivity analysis: adjusted odds ratios of ENT diagnoses controlling individually for Charlson Comorbidity Index elements.

| **Charleson Comorbidity Index** | **ENT Diagnosis** | | | | | | | | |
| --- | --- | --- | --- | --- | --- | --- | --- | --- | --- |
|  | **Dysphagia  (OR 95% CI)** | **Dysphonia**  **(OR 95% CI)** | **Chronic Rhinitis**  **(OR 95% CI)** | **Chronic Sinusitis**  **(OR 95% CI)** | **Vocal Cord Paralysis**  **(OR 95% CI)** | **Salivary Gland Pathologies**  **(OR 95% CI)** | **Cranial Nerve Pathologies**  **(OR 95% CI)** | **Epistaxis**  **(OR 95% CI)** | **Impacted Cerumen**  **(OR 95% CI)** |
| **Congestive Heart Failure** | *2.010  (1.780-2.270)** | *2.242  (1.873-2.683)** | *2.774  (2.400-3.206)** | 2.328  (2.062-2.629)* | *2.659  (1.763-4.009)** | 0.840  (0.606-1.167) | *2.417  (1.859-3.143)** | *1.798  (1.477-2.189)** | *0.596  (0.478-0.744)** |
| **Dementia** | *2.547  (2.268-2.860)** | *2.536  (2.136 - 3.011)** | *3.075  (2.677 - 3.533)** | 2.503  (2.226-2.814)* | *3.250  (2.200-4.799)** | 0.896  (0.659 - 1.218) | *2.628  (2.270-3.043)** | *2.528  (2.100 -3.043)** | *0.677  (0.547-0.838)** |
| **Diabetes Mellitus with Complications** | *2.329  (2.071-2.619)** | *2.419  (2.033-2.878)** | *2.962  (2.574-3.408)** | 2.402  (2.134-2.703)* | *3.048  (2.054-4.523)** | 0.849  (0.623-1.158) | *2.488  (2.145-2.886)** | *2.304  (1.909-2.78)** | *0.639  (0.515-0.793)** |
| **Hemiplegia** | *2.353  (2.091-2.649)** | *2.216  (1.857-2.643)** | *2.967  (2.58-3.413)** | 2.459  (2.186-2.766)* | *1.542  (1.013-2.345)** | 0.87 (0.638-1.186) | *2.224  (1.904-2.597)** | *2.472  (2.05-2.98)** | *0.676  (0.546-0.838)** |
| **HIV/AIDS** | *2.583  (2.302-2.898)** | *2.577  (2.171-3.059)** | *3.103  (2.701-3.564)** | 2.535  (2.256-2.849)* | *3.323  (2.252-4.903)** | 0.899  (0.661-1.222) | *2.659  (2.297-3.078)** | *2.56  (2.127-3.080)** | *0.684  (0.553-0.847)** |
| **Malignancy** | *2.114  (1.877-2.381)** | *2.001  (1.679-2.384)** | *2.504  (2.172-2.886)** | 2.076  (1.840-2.341)* | *2.542  (1.714-3.772)** | *0.707  (0.518-0.964)** | *2.277  (1.961-2.643)** | *2.084  (1.726-2.517)** | *0.549  (0.442-0.681)** |
| **Metastatic Solid Tumor** | *2.303  (2.048-2.591)** | *2.216  (1.861-2.639)** | *2.809  (2.440-3.234)** | 2.321  (2.062-2.613)* | *2.904  (1.957-4.311)** | 0.792  (0.580-1.080) | *2.457  (2.118-2.851)** | *2.292  (1.899-2.766)** | *0.608  (0.490-0.754)** |
| **Mild Liver Disease** | *2.229  (1.980-2.509)** | *2.211  (1.855-2.636)** | *2.749  (2.386-3.168)** | 2.295  (2.037-2.585)* | *3.034  (2.039-4.514)** | 0.765  (0.554-1.056) | *2.396  (1.855-3.095)** | *2.179  (1.802-2.634)** | *0.637  (0.513-0.790)** |
| **Moderate/Severe Liver Disease** | *2.547  (2.269-2.859)** | *2.581  (2.174-3.065)** | *3.101  (2.699-3.563)** | 2.778  (1.976-3.905)* | *3.344  (2.266-4.937)** | 0.889  (0.647-1.221) | *2.742  (2.134-3.522)** | *2.467  (2.047-2.973)** | *0.691  (0.558-0.856)** |
| **Chronic Pulmonary Disease** | *1.829  (1.62-2.065)** | *1.74  (1.456-2.080)** | *2.084  (1.802-2.410)** | 1.695  (1.499-1.918)* | *2.271  (1.518-3.398)** | *0.702  (0.512-0.963)** | *2.265  (1.944-2.639)** | *1.767  (1.458-2.142)** | *0.574  (0.461-0.715)** |
| **Renal Disease** | *2.048  (1.817-2.309)** | *2.132  (1.787-2.544)** | *2.722  (2.36-3.138)** | 2.197  (1.949-2.477)* | *2.616  (1.753-3.905)** | 0.795  (0.581-1.087) | *2.383  (2.051-2.77)** | *1.968  (1.626-2.381)** | *0.598  (0.481-0.744)** |
| **Rheumatic Disease** | *2.135  (1.893-2.408)** | *2.173  (1.817 - 2.599)** | *2.629  (2.276 - 3.038)** | 2.154  (1.908-2.432)* | *2.721  (1.809 -4.093)** | *0.647  (0.469 - 0.892)** | *2.339  (2.009-2.723)** | *2.091 (1.722-2.539)** | *0.642  (0.516 -0.800)** |
| ****- p-value < .05*** | | | | | | | | | |
